# Supplementary material for: Levels of and changes in socioeconomic inequality in delivery care service: A decomposition analysis using Bangladesh Demographic Health Surveys
Source: PLoS One. 2020 Nov 30;15(11):e0242325. doi: 10.1371/journal.pone.0242325 (PMC7703934; doi:10.1371/journal.pone.0242325)
Supplement: Attachment — Submitted filename: Review of the Manuscript-PONE-D-19-33387.docx [file pone.0242325.s001.docx]

**Manuscript:** Levels of and changes in socioeconomic inequality in delivery care service utilization in Bangladesh.

**Summary**

The authors explored Levels of and changes in socioeconomic inequality in delivery care service utilization in Bangladesh using nationally representative data from DHS carried out in 2011 and 2014. The idea sounds very interesting as such kinds of analyses can guide policymakers to understand where most efforts are necessary to reduce gap between subgroups and reach universal health coverage in delivery care as well as to improve maternal health services. However, there are gaps and concerns in the methodology that need to be addressed.

**Major issues**

1. The last two recent surveys in Bangladesh were carried out in 2012 (Multiple indicators cluster surveys-MICS) and in 2014 (DHS). Since the authors used the last two recent DHS, is there any reason to use the 2011-DHS instead of the one conducted in 2012 (MICS-2012)?
2. As the authors aimed to assess changes in inequality overtime, another concern is: Why the authors preferred to use only two DHS (2011 and 2014) while a series of other surveys including MICS and DHS were carried out in Bangladesh before 2014? Is this period sufficient to capture change in inequality overtime? I strongly recommend the authors to include in their analyses other national surveys carried in the country before 2011.
3. A recent study conducted by Khan et al, which was also cited in this manuscript performed the same decomposition analysis using DHS (2004 and 2014) and C-section as outcome. Could the authors highlight what this study add to the literature once a similar study using the same DHS and the outcome (c-section) was already published (**see*: Khan MN, Islam MM, Rahman MM. Inequality in utilization of cesarean delivery in Bangladesh: a decomposition analysis using nationally representative data. Public Health. 2018;157:111–20****).*
4. Page 12- line 137: Move this variable of socioeconomic status to the subsection of covariates and provide more details on PCA technique as well as examples of the assets used to calculate the wealth index. Likewise, more details are necessary about the process of calculating the wealth index and explain what the first and the last quintiles meant.
5. In Page 17- from line 219 to 231. The estimates presented by the authors must be interpreted with caution as the authors performed only crude analyses using logistic regression. I would suggest the authors to perform a multivariate logistic regression analysis and assess the effect of such covariates on the outcomes.

**Minor issues**

1. Title: The design of the study should be mentioned in the title.
2. Page 9-Line 68-70: Where it is said “***The Sustainable Development Goals (SDGs) aims to reduce global maternal mortality to less 75 than 70 deaths per 100,000 live births and to reduce the incidence of neonatal and infant deaths76 to as low as 12 and 25 deaths per every 1,000 live births, respectively****”.* Please include the deadline. For example, until 2025, 2030, etc…
3. Page 9-line 77-79: Specify whether such countries are low, middle- or high-income countries.
4. Page 9-line 80: Where it is said “***Bangladesh has remarkably improved.*** Please provide references.
5. Page 11-line 116-125: It is necessary to include a sentence regarding the local institution responsible for collecting the information on ethical issues.
6. Page 11-line 132-133: The authors should provide a clear definition of medically trained professional, which categories of professionals are considered as trained and explain whether such definitions was kept in both surveys used.
7. Page 12-line 146: Specify whether age, religion, education, etc… used in this study were for women, husband, children, etc.…
8. Page 14 from line 201-201, the authors provided an equation about the decomposition analysis which I consider very interesting. However, it is not clear in the section of results whether the authors performed a crude or adjusted analysis and how the covariates were treated into the model.
9. Page 15-line 207-208: mention also coverage for the other outcomes used and respectively for both years (2011 and 2014).
10. From Table 2 onwards, I consider that results for skilled birth attendant could be placed in appendix since other previous study showed high correlation between SBA and institutional delivery (see: ***Joseph G, da Silva IC, Wehrmeister FC, Barros AJ, Victora CG. Inequalities in the coverage of place of delivery and skilled birth attendance: analyses of crosssectional surveys in 80 low and middle-income countries. Reprod Health. 2016;13(1):77***).
11. Table 2: The authors could add other columns on the right side of the SE and include the p value for each covariate in relation to the outcomes, which would help to delete all those Asterix included in the Table. Besides, it is not necessary to put an Asterix for each estimate on the Table.
12. Page 32 in Figure 1, I recommend the authors to reconsider the Y scale (from zero to 100%) instead of the one used.
13. Page 33. Figure 2 can be deleted as it brought no new information when compared to Figure 1.
14. Page 15 Table 4 seems confusing and it is not clear what the authors meant by those estimated presented.
