# Supplementary material for: Levels of and changes in socioeconomic inequality in delivery care service: A decomposition analysis using Bangladesh Demographic Health Surveys
Source: PLoS One. 2020 Nov 30;15(11):e0242325. doi: 10.1371/journal.pone.0242325 (PMC7703934; doi:10.1371/journal.pone.0242325)
Supplement: Attachment — Submitted filename: Review_Response_R1.docx [file pone.0242325.s002.docx]

**Levels of and changes in socioeconomic inequality in delivery care service****:** **A decomposition analysis using Bangladesh Demographic Health Surveys**

**Response to reviewers’ comments**

**Journal Requirements:**

When submitting your revision, we need you to address these additional requirements.

1. Please ensure that your manuscript meets PLOS ONE's style requirements, including those for file naming.

Response: We have made every effort so that our revised manuscript meets PLOS ONE's style requirements.

2. In your Methods section, please provide additional information on how the wealth index was calculated.

Response: We have added the information to calculate the wealth index in the method section.

3. Our internal editors have looked over your manuscript and determined that it is within the scope of our Health Inequities and Disparities Research Call for Papers. This collection of papers is headed by a team of Guest Editors for PLOS ONE: Clare Bambra, Hans Bosma, Diana Burgess, Joseph Telfair, Barbara Turner, and Jennie Popay. The Collection will encompass a diverse range of research articles on health inequities and disparities. Additional information can be found on our announcement page: <https://collections.plos.org/s/health-inequities>. If you would like your manuscript to be considered for this collection, please let us know in your cover letter and we will ensure that your paper is treated as if you were responding to this call. If you would prefer to remove your manuscript from collection consideration, please specify this in the cover letter.

Response: Thanks, we would like our manuscript to be considered for this collection.

Additional Editor Comments (if provided):

Given that the purpose of the study is to investigate changes in inequality over time, I agree with the second reviewer's comment that the period from 2011 to 2014 is too short and his suggestion to include in your analysis other national surveys carried out prior to 2011.

Response: To our knowledge, there is no theoretical or empirically established timeframe for studying changes in inequalities. We would appreciate if the reviewer could enlighten us on this issue. Our study aims to examine socioeconomic inequality in delivery care services which is affected by socioeconomic factors. Although our study period was short, there was a substantial change in the distribution of socioeconomic factors between this period. We have responded to second reviewer's comment in details on this matter.

**Reviewer #1:**

Major points:

1. The article’s message hinges on the choice of index (particularly between EI and WI)- while EI shows an increase over the study period the other two indices show a decline. The authors defend EI with their argument but it will be a good idea to mention a critique to their argument, as suggested in the paper below:

Kjellssona & Gerdthama, 2011. Correcting the Concentration Index for Binary Variables. Retrieved from https://project.nek.lu.se/publications/workpap/papers/WP11_4.pdf on April 2, 2020

I have pasted excerpts of the relevant text from pages 19-20:

“If the localization of the threshold within the distribution of the latent variable (compare Figure 5) is due to either arbitrariness or cultural differences, we argue that level independence is a desirable property; given that the level of prevalence is due to an arbitrary threshold, it is sensible if the measured degree of inequalities is invariant to the level of prevalence. ….

By contrast, the threshold for a diagnosed medical condition, e.g. diabetes, is less subjective and there is less variation between contexts. The prevalence of diabetes can therefore be considered to be both accurate and interesting information, and thus an analysis of relative inequalities may be appropriate. In such a case, level independence is not necessarily desirable and, given the normative acknowledgment of the mirror relativity of W, the choice of index should depend on the preferred value judgment. Accordingly, we can sum up the discussion in a two by two matrix (see Figure 6). If the threshold of the latent variable is arbitrary or subjective, i.e. if there is a risk of reporting heterogeneity, one should consider using E. In turn, if the threshold is objective, the choice ought to depend on the imposed value judgment.” The three indicators of delivery care belong to an objective category. The two indices differ mainly on the property of level independence and the choice of index is based on the “preferred value judgement”. The latter should be clarified, in explicit terms, in the text.

Response: We would like to clarify that there was no statistically significant difference between the estimates of the WI in two periods. Therefore, we could not say that inequality declined as measured by the WI. We have removed the estimates using the CI as we believe that it is not appropriate to report these estimates when comparing inequalities between two periods. This could be misleading for the readers. We agree with the reviewer that there are arguments against using the EI. However, the discussion on the preferred value judgement is more theoretical and still debated in the literature. This discussion is beyond the scope of our paper. We have added two sentences in the method section to clarify this issue. The indicators of delivery care belong to an objective category, but the data are self-reported. So, the indicators might be subject to re-call bias.

2. Please clarify the age-distribution in the data-sets. The 2011 Bangladesh DHS surveyed women from ages 13 years onward while 2014 Bangladesh DHS surveyed women from ages 15 years onward. The correction will most likely change the estimates for 2011.

Response: Thanks for raising this issue. In 2011, only 0. 5% women aged below 15 years were interviewed. We included women with age 15+ years in the analysis for 2011 to keep both surveys comparable.

3. The discussion section lacks clarity and needs to be strengthened. A major point of the study was to use EI instead of CI and WI but the authors have not touched on this point at all in the discussion. It would have been a good idea to expand on how the difference in calculations gives more insight into the changes in inequality over time. For example, the major recipients of delivery care in 2014 must have been in the richest 50% population (denominator of EI) thereby showing an increased EI but not in the richest 10% population (denominator of WI) thereby showing a decreased WI. The study shows an increase in EI over time, but this is due to changes in sensitivity to certain predictors and not due to changes in CI of the said predictors (which in fact show a decline). The authors fail to clearly demarcate this and explain or theorize on why this is so. Additional points below.

Response: We have included a paragraph in the discussion to address this comment.

**Some minor points:**

Lines 129-132: The description of health facility delivery: While there are no ‘health posts’ in the variable description of m15_1 in Bangladesh DHS 2011 or 2014, categories like upazila health & family welfare center; other public sector; community clinic; and other ngo sector have not been included in the description. Does it mean that these go into the “0” category?

Response: Thanks for identifying this mistake. We have now deleted “health posts” from the description. We have revised the description of health facility delivery.

Line 133: It will be a good idea to detail the categories included as ‘a medically trained professional’.

Response: Thanks for this suggestion. We have now included the categories of a medically trained professional.

Line 247: Do the predicted probabilities refer to those obtained following the multivariate logistic regression? Please clarify.

Response: We obtained the predicted probabilities from the multivariate logistic regression model with interaction between survey year and wealth index. We have revised the text to clarify this.

Lines 249-251: That is a sweeping statement and does not apply to skilled care for the 2nd and 4th wealth quintile.

Response: We have revised the text to correct this mistake.

Lines 268-276: I will like to understand why the relative importance of 4+ANC visits decreased. The same goes for the relative importance of household wealth, women’s education, and husband’s education. What does it signify? Were there some policy changes or some other ground realities that could be behind these findings?

Response: The relative importance of 4+ ANC visits decreased because there was a reduction in inequality of 4+ ANC visits and the strength of association between delivery care use and 4+ ANC visits also decreased. If there were no changes in inequality in other factors affecting delivery care services, we would expect overall inequality to be decreased. However, it was not case as we have seen in the decomposition results. There were no explicit policy changes between the study periods, to which such changes can be attributed.

Table 4 also raises the same questions as above. While we observe a reduction in inequality for independent factors (in 2nd & 3rd columns in Tables 4) like 4+ ANC visits, women’s and husband’s education (except for primary education), and in household wealth (except for the poorer), the relative contribution of these has changed in the models predicting the three delivery care indicators. Why? The explanation in “Lines 268-276” suggests substitution but again, the question is why?

Response: The relative contribution of these variables has changed because the association of these variables became strong in 2014.

Table 5: The above is further corroborated by the findings in Table 5. Here again, the findings suggest that the change in CI is mainly due to higher sensitivity to the parameters. On the other hand, the change in CI contributes negatively meaning that the direction of change for household wealth, women’s and husband’s education is the same as 4+ ANC visits. The discussion section should explain what this signifies.

Response: Thank you for your comment. As you point it out, in our case, the changes in the CI of these variables indicate the negative contributions. Given that they are positively related with the outcome variables, their negative contributions are attributable to the reductions in their respective socioeconomic inequalities. This suggests that the mitigation of the socioeconomic inequalities of these determinants of delivery care use over time partially alleviates the deteriorating inequality in maternal healthcare use.

Lines 324-325 & Lines 334-337: The authors refer to the same article for contradictory views, namely “reduction in inequality of delivery care services” and “increasing socioeconomic inequality”.

Response: Thank you very much for identifying this contradiction. We have deleted line 334-337 from the revised version to address this comment.

Lines 337-339: How did the authors reach this conclusion?

Response: Thanks for refereeing to misleading conclusion. We have deleted these lines from the revised version.

Lines 348-350: Please elaborate on this sentence.

Response: We have revised the text in the manuscript.

Lines 353-354: 4+ ANC visits actually caused a reduction in CI from 2011 to 2014. When the authors say that it has contributed to inequality, I guess they refer to the positive CI in the 2nd & 3rd columns in Tables 4 (these are independent CI for each predictor for 2011 and 2014). This causes unnecessary confusion because so far, the narrative was about decomposition of inequality explained by a particular predictor within the multivariate model and its change over time.

Response: We have edited the text follow this comment.

Lines 387-388: How did the authors come to this conclusion? How and when was need defined?

Response: We have revised this section to address reviewer’s concern.

**Reviewer #2:**

**Summary**

The authors explored Levels of and changes in socioeconomic inequality in delivery care service utilization in Bangladesh using nationally representative data from DHS carried out in 2011 and 2014. The idea sounds very interesting as such kinds of analyses can guide policymakers to understand where most efforts are necessary to reduce gap between subgroups and reach universal health coverage in delivery care as well as to improve maternal health services. However, there are gaps and concerns in the methodology that need to be addressed.

**Major issues**

1. The last two recent surveys in Bangladesh were carried out in 2012 (Multiple indicators cluster surveys-MICS) and in 2014 (DHS). Since the authors used the last two recent DHS, is there any reason to use the 2011-DHS instead of the one conducted in 2012 (MICS-2012)?

Response: Thanks for raising this issue. We would like to clarify that the explanatory variables used in this study are not comparable in MICS and DHS. This would preclude us to examine the effects of changes in the distributions of important predictors of delivery care use by applying the Oaxaca-Blinder decomposition method.

2. As the authors aimed to assess changes in inequality overtime, another concern is: Why the authors preferred to use only two DHS (2011 and 2014) while a series of other surveys including MICS and DHS were carried out in Bangladesh before 2014? Is this period sufficient to capture change in inequality overtime? I strongly recommend the authors to include in their analyses other national surveys carried in the country before 2011.

Response: There are several reasons to choose 2011 and 2014 BDHSs. For example, in the surveys before 2011, data on delivery care services use were collected for the live births five year preceding the survey. From 2011, data were collected for live births in the three years preceding the survey. In addition, variable definition and categories should remain same in both surveys to implement the decomposition analysis. Otherwise, the results are not comparable between two periods. As as an example, there were six regions before 2011 in Bangladesh. In fact, Khan et al (2018) did not consider this issue in their study. Moreover, there was a notable change in the average and distribution of outcomes variables between 2011 and 2014. Therefore, the study period was sufficient to capture change in inequality overtime. There exist some studies in the literature which used brief period to understand the changes in inequality. An example is:

Dorjdagva J, Batbaatar E, Dorjsuren B, Kauhanen J. Income-related inequalities in health care utilization in Mongolia, 2007/2008–2012. International Journal for Equity in Health. 2015 Dec;14(1):1-0.

3. A recent study conducted by Khan et al, which was also cited in this manuscript performed the same decomposition analysis using DHS (2004 and 2014) and C-section as outcome. Could the authors highlight what this study add to the literature once a similar study using the same DHS and the outcome (c-section) was already published (**see*: Khan MN, Islam MM, Rahman MM. Inequality in utilization of cesarean delivery in Bangladesh: a decomposition analysis using nationally representative data. Public Health. 2018;157:111–20****).*

Response: Our paper differs on several respects. First, the study by Khan et al (2018) has a few methodological limitations. We have mentioned these limitations at the end of introduction in the revised version of the manuscript. Second, whilst Khan et al 2018 used only one variable, as you have read, we have used two other important indicators of delivery care services. Therefore, our study offers a complete picture of socioeconomic inequalities in delivery care services in Bangladesh.

4. Page 12- line 137: Move this variable of socioeconomic status to the subsection of covariates and provide more details on PCA technique as well as examples of the assets used to calculate the wealth index. Likewise, more details are necessary about the process of calculating the wealth index and explain what the first and the last quintiles meant.

Response: Thanks for this suggestion. We have revised the text to include a description of the PCA method.

5. In Page 17- from line 219 to 231. The estimates presented by the authors must be interpreted with caution as the authors performed only crude analyses using logistic regression. I would suggest the authors to perform a multivariate logistic regression analysis and assess the effect of such covariates on the outcomes.

Response: We would like to clarify that our results and related interpretations are based on multivariate logistic regression. We have revised the title of Table 2 to clarify this.

**Minor issues**

1. Title: The design of the study should be mentioned in the title.

Response: The title now includes the design of the study.

2. Page 9-Line 68-70: Where it is said “***The Sustainable Development Goals (SDGs) aims to reduce global maternal mortality to less 75 than 70 deaths per 100,000 live births and to reduce the incidence of neonatal and infant deaths 76 to as low as 12 and 25 deaths per every 1,000 live births, respectively****”.* Please include the deadline. For example, until 2025, 2030, etc…

Response: We have included the deadline in the introduction.

3. Page 9-line 77-79: Specify whether such countries are low, middle- or high-income countries.

Response: We have specified the categories of countries in the revised version.

4. Page 9-line 80: Where it is said “***Bangladesh has remarkably improved.*** Please provide references.

Response: Thanks for pointing this issue. We have added a reference.

5. Page 11-line 116-125: It is necessary to include a sentence regarding the local institution responsible for collecting the information on ethical issues.

Response: We have added ethics statement at the end of method section.

6. Page 11-line 132-133: The authors should provide a clear definition of medically trained professional; which categories of professionals are considered as trained and explain whether such definitions was kept in both surveys used.

Response: We have included the categories of a medically trained professional and explained whether such definitions was kept in both surveys used.

7. Page 12-line 146: Specify whether age, religion, education, etc… used in this study were for women, husband, children, etc.…

Response: We have revised this section as suggested.

8. Page 14 from line 201-201, the authors provided an equation about the decomposition analysis which I consider very interesting. However, it is not clear in the section of results whether the authors performed a crude or adjusted analysis and how the covariates were treated into the model.

Response: All the analyses from Table 2 and onwards are adjusted.

9. Page 15-line 207-208: mention also coverage for the other outcomes used and respectively for both years (2011 and 2014).

Response: Revised.

10. From Table 2 onwards, I consider that results for skilled birth attendant could be placed in appendix since other previous study showed high correlation between SBA and institutional delivery (see: ***Joseph G, da Silva IC, Wehrmeister FC, Barros AJ, Victora CG. Inequalities in the coverage of place of delivery and skilled birth attendance: analyses of crosssectional surveys in 80 low and middle-income countries. Reprod Health. 2016;13(1):77***).

Response: We certainly agree with reviewer that there is a high correlation between SBA and institutional delivery. The paper referred by the reviewers is a multicounty and descriptive study where the authors presented several statistics. On the other hand, our study particularly focuses on detail analysis of inequalities in delivery care services in Bangladesh. We also find some differences in results between facility delivery and skilled birth attendance. In addition, inclusion of skilled birth outcome does not need additional table in the main text. Therefore, we respectfully disagree with the reviewer on this point.

11. Table 2: The authors could add other columns on the right side of the SE and include the p value for each covariate in relation to the outcomes, which would help to delete all those Asterix included in the Table. Besides, it is not necessary to put an Asterix for each estimate on the Table.

Response: We revised Table 2 and included p-values as suggested by the reviewer.

12. Page 32 in Figure 1, I recommend the authors to reconsider the Y scale (from zero to 100%) instead of the one used.

Response: We have edited Figure 2 following your suggestion.

13. Page 33. Figure 2 can be deleted as it brought no new information when compared to Figure 1.

Response: We respectfully disagree with the reviewer on this point. Figure 2 is different from Figure 1. Figure 2 provides further insight on the inequality it plots predicted probabilities from multivariate regression models with interaction between year and the wealth index. We have revised the text to makes the importance of Figure 2 clear.

14. Page 15 Table 4 seems confusing and it is not clear what the authors meant by those estimated presented.

Response: Table 4 is central to our study. It presents the factors contributing to wealth-related inequalities in delivery care services. We have revised the title of Table 4 to address reviewer’s concern.
