# Supplementary material for: Levels of and changes in socioeconomic inequality in delivery care service: A decomposition analysis using Bangladesh Demographic Health Surveys
Source: PLoS One. 2020 Nov 30;15(11):e0242325. doi: 10.1371/journal.pone.0242325 (PMC7703934; doi:10.1371/journal.pone.0242325)
Supplement: Attachment — Submitted filename: R2_Response to Reviewer.docx [file pone.0242325.s003.docx]

**Levels of and changes in socioeconomic inequality in delivery care service: A decomposition analysis using Bangladesh Demographic Health Surveys**

**PONE-D-19-33387R1**

**Response to reviewers’ comments**

**Additional Editor Comments (if provided):**

The manuscript has been re-reviewed by one of the reviewers that commented on the first version. Note that one of her suggestions -- incorporating the 2018 DHS data into the analysis -- has since been withdrawn, as it was determined that the 2018 dataset is not yet publicly available. However, there are still a few improvements that need to be made to the manuscript before we issue a decision to accept it for publication. Please review the reviewer's suggestions to modify the methodology section to reflect that CI is no longer presented in the study -- as well as the other comments and suggestions. I look forward to receiving a revised version of the manuscript, along with point by point responses.

Response: Thank you very much for the positive evaluation of our article. We have addressed the comments from the reviewer on the revised version.

**Reviewer #1:**

The authors have done a good job in explaining some of their results. However, there is still quite a bit of confusion in the corrected version owing to the removal of CI results, taking a stand on EI as opposed to WI and justifying its use, and use of some incorrect words. Also, after reading the other reviewer’s and the editor’s comments, I will like to point out that the 2018 Bangladesh DHS is available. It seems odd that the authors have not used it.

Response: Thanks for your positive feedback on the revised version.

- If authors have removed the CI, then why do they still describe it in Methods section (Line 173 onwards)? Given that the authors have removed all initial results on CI, it does not make sense to then provide a decomposition of CI. If it is decomposition of EI, then table headings need to be corrected.

Response: We have decomposed the EI. We would like to clarify that inequality by factor contributions are measured by the CI despite of the decomposition of the EI. Please see equation 4 for this clarification. We have now changed the table headings to follow this comment.

- It seems that the authors do not think that WI is useful (Lines 189-190 & 198-199). If the authors’ preference is EI (Lines 199-200), the need of explaining and estimating WI seems superfluous. Accordingly, a lot of unnecessary technical detail in the methods section can be removed and an explanation about EI and why it has been chosen should be enough.

Response: We removed the technical details of the WI to be consistent in the paper.

- The authors refer to WI as relative inequality in Lines 363-364 while they have earlier explained WI as “WI is neither an absolute nor a relative measure of inequality” (Lines 199-200).

Response: We have now removed all mentions of the WI.

- Should the word be “decomposed” in Lines 185, 188, and 197? These refer to the corrections made to CI for estimation of WI and EI.

Response: We have changed the CI to the EI. Please see line 189 in the revised version. We have carefully checked line 188 and 197 and we confirm these are correct.

- The sentence in Lines 453-455 needs to be rechecked. This is because the declining inequality trend in last decade was probably measured using CI which even now shows a similar trend (though results have been removed). Also, the authors should be careful in using the words equity and inequity as opposed to equality and inequality.

Response: Thanks for this suggestion. We have revised the conclusion based on this comment.

- I will suggest the use of ‘skilled birth attendance’ instead of ‘skilled delivery’.

Response: Thanks for this suggestion. We have revised the paper following this suggestion.
